# *CYP2D6* Phenotyping Using Urine, Plasma, and Saliva Metabolic Ratios to Assess the Impact of *CYP2D6^∗^10* on Interindividual Variation in a Chinese Population

**DOI:** 10.3389/fphar.2017.00239

**Published:** 2017-05-02

**Authors:** Rui Chen, Xin Zheng, Pei Hu

**Affiliations:** Clinical Pharmacology Research Center, Peking Union Medical College HospitalBeijing, China

**Keywords:** *CYP2D6^∗^10*, phenotyping method, metabolic ratio, polymorphism, genotype

## Abstract

**Purpose:** Asian populations have around 40–60% frequency of reduced function allele *CYP2D6^∗^10* compared to 1–2% in Caucasian populations. The wide range of *CYP2D6* enzyme activities in subjects with the *CYP2D6^∗^10* variant is a big concern for clinical practice. The quantitative analysis measuring the impact of *CYP2D6* enzyme activity as a result of one *CYP2D6^∗^10* allele or two *CYP2D6^∗^10* alleles has not been reported in large Asian populations.

**Methods:** A total of 421 healthy Chinese subjects were genotyped for *CYP2D6* by polymerase chain reaction and direct DNA sequencing. A total of 235 subjects with *CYP2D6^∗^1/^∗^1* (*n* = 22), *CYP2D6^∗^1/^∗^10* (*n* = 93), *CYP2D6^∗^10/^∗^10* (*n* = 85), and *CYP2D6^∗^5/^∗^10* (*n* = 35) were phenotyped for *CYP2D6* using dextromethorphan as the probe drug. Metabolic ratios (MR) were calculated as the ratio of parent drug to metabolite in 0–3 h urine, 3 h plasma, and 3 h saliva for each sample type.

**Results:** The urinary, plasma, or salivary MRs increased successively in subjects with *CYP2D6^∗^1/^∗^1*, *^∗^1/^∗^10*, *^∗^10/^∗^10*, and *^∗^5/^∗^10* (all *P* < 0.001). In the normal metabolizer group, homozygous *CYP2D6^∗^10/^∗^10* decreased the *CYP2D6* enzyme activity further than heterozygous *CYP2D6^∗^1/^∗^10*. Urinary, plasma, and salivary MRs were highly correlated.

**Conclusion:** The normal metabolizer group calls for a more detailed classification. The activity score system could more accurately predict enzyme activity than by grouping a number of genotypes into a single phenotype group. Single-point plasma samples and saliva samples could be used as alternative phenotyping methods for clinical convenience.

## Introduction

The *CYP2D6* gene encodes the cytochrome P450 2D6 enzyme that is a member of the cytochrome P450 superfamily. It plays an important role in the metabolism of approximately 25% of currently marketed drugs. This enzyme participates in metabolizing a number of substrates in the therapeutic class of clinical drugs including antidepressants, antipsychotics, analgesics and antitussives, beta adrenergic blocking agents, antiarrhythmic, antiemetics, etc. (Zhou, 2009a,b; Murphy and McMahon, 2013; Zanger and Schwab, 2013). The *CYP2D6* gene product is also one of the most famous and widely investigated polymorphic enzymes due to its broad inter-individual and inter-ethnic variations in enzyme activity. This led to the discovery of mutation, deletion, and duplication variants of the *CYP2D6* gene (Teh and Bertilsson, 2012). Such diverse enzyme activities have been shown to result in dose-dependent adverse events or therapeutic failures after administration of *CYP2D6* substrates (Gaedigk, 2013).

Currently, there are more than 100 allelic variants of *CYP2D6* identified^1^. Among these are full functional alleles, reduced function alleles, non-functional alleles and gene copy duplicates, that range in activity from ultra-rapid metabolism to no metabolism (Gaedigk, 2013; Hicks et al., 2016; Gaedigk et al., 2017). The frequencies of *CYP2D6* alleles can vary dramatically among ethnicities. For example, non-functional *CYP2D6^∗^4* allele frequency can be extremely low or even absent in some east Asian and Oceania populations while Europeans frequencies typically range between 15 and 20% but can be over 30% as reported in people of Faroese decent (Gaedigk, 2013). In contrast, on a population basis, Asians exhibit a marked shift toward overall slower *CYP2D6* activity when comparing metabolic ratios (MR) from urine samples (Kitada, 2002) that can be presented as high frequencies (up to 64%, averaging 42%) of the reduced function allele *CYP2D6^∗^10* (Gaedigk, 2013). In other populations the frequencies of *CYP2D6^∗^10* range between 3 and 7% and the frequency is the lowest in white Europeans (Gaedigk, 2013) and Oceanians (Gaedigk et al., 2017).

Phenotypes are stratified into groups including poor (PM), intermediate (IM), normal (NM), and ultra-rapid (UM) metabolizer phenotypes (Gaedigk et al., 2008; Caudle and Dunnenberger, 2017). The activity score (AS) system for *CYP2D6* was introduced by Gaedigk et al. (2008) and was used to translate diplotypes into predicted phenotypes (Hicks et al., 2016). Metabolic activity of *CYP2D6* is assessed by probe drugs, where dextromethorphan is the most often used substrate. Eight-hour urinary MR of dextromethorphan (DM) to its metabolite dextrorphan (DX) is employed to differentiate between NMs and PMs (Chladek et al., 2000; O’mathúna et al., 2008; Lötsch et al., 2009; Ito et al., 2010). However, collection of urine during the 8 h interval is a demanding process and inconvenient for clinical operation. Therefore, alternative procedures have been developed for simple and robust phenotyping. In addition to urine, plasma, or saliva samples can also be used to determine MR. Previous studies indicated tight correlations between MRs measured in plasma (3 h post-dose) and urine (0–4 h post-dose) (Chladek et al., 2000). The MR from single-point plasma 1 to 30 h post-dose was reported to have good correlations with MR from area under the curve (AUC) (Chen et al., 2016a,b). The MR from plasma collected 3, 4, or 6 h post-dose and MR from saliva collected 2, 3, 4, 5, 6 h post-dose were explored to discriminate between NM from PM and IM (Frank et al., 2007). These results showed that single-point plasma and saliva-derived MRs may serve as alternative tools for *CYP2D6* phenotyping.

It has been shown that the *CYP2D6^∗^10* variant is a reduced function allele that decreases enzyme activity and thus increases the *CYP2D6* MR value. However, quantitative analysis on the impact of *CYP2D6* enzyme activity as a result of one *CYP2D6^∗^10* allele or two *CYP2D6^∗^10* alleles has not been reported in large populations. In the present study, *CYP2D6^∗^1* was defined as a standard full functional allele and *CYP2D6^∗^5* was defined as a non-functional allele. Both *CYP2D6^∗^1* and *CYP2D6^∗^5* were used as references or control alleles to investigate the impact of the *CYP2D6^∗^10* allele on the metabolic activity of *CYP2D6* in a healthy Chinese population. The *CYP2D6* enzyme activities within the population were determined simultaneously by urinary, plasma, and salivary phenotyping methods and the results were compared.

## Materials and Methods

### Study Subjects

The study was performed in accordance with the Declaration of Helsinki and was approved by the Ethics Committee of Peking Union Medical College Hospital. Written informed consent was obtained from each subject. Four hundred and twenty-one healthy unrelated subjects in Mainland China were enrolled. All enrolled subjects were judged to be healthy based on the results of detailed physical examination, 12-lead electrocardiography, biochemistry, hematology, and routine urinalysis. Subjects were not eligible if they had history or evidence or hepatic, renal, gastrointestinal, or hematologic abnormality; hepatitis B or C, syphilis, or human immunodeficiency virus infection on screening examination; any other acute or chronic disease; or allergic to dextromethorphan. The consumption of alcohol, grapefruit juice, and caffeine-containing drinks was not permitted for 24 h prior to DM administration and until all samples were collected in the study phase. The subjects were instructed to abstain from taking any medication or herbal remedies for at least 1 week and smoking for at least 3 days before the study.

### *CYP2D6* Genotyping

Peripheral blood samples from 421 subjects were collected and DNA was extracted by total genomic DNA isolation using a Wizard TM Genomic Purification Kit (Promega, USA). The DNA from 2 mL of blood was dissolved in 100 μL of DNA hydration solution and stored at -70°C. All subjects recruited in this study were genotyped by DNA sequencing analysis for *CYP2D6^∗^1, ^∗^2, ^∗^3, ^∗^4, ^∗^6, ^∗^7, ^∗^10, ^∗^14, ^∗^18, ^∗^21, ^∗^28, ^∗^33, ^∗^34, ^∗^35, ^∗^36, ^∗^39, ^∗^41, ^∗^43, ^∗^49, ^∗^51, ^∗^52, ^∗^54, ^∗^60, ^∗^63, ^∗^65, ^∗^69, ^∗^71*, and *^∗^75* as previously reported (Qian et al., 2013). Long polymerase chain reaction (PCR)-based methods from Løvlie et al. (1996) and Hersberger et al. (2000) were employed with minor modifications to detect the *CYP2D6^∗^5* allele and duplication, respectively. We tested the aforementioned alleles and duplications to determine *CYP2D6^∗^1, ^∗^5*, and *^∗^10* specifically. The *CYP2D6* gene was amplified using a standard procedure (Qian et al., 2013). *CYP2D6* gene sequencing had the entire gene coverage as well as 5′ and 3′ UTR regions corresponding to M33388 positions -2182 to 4482. Primers for each fragment of gene were designed using Primer Specification Design v1.1 (Capitalbio, China) (Supplementary Table 1). Fragments were amplified by PCR. To confirm PCR products sizes, samples were electrophorezed on a 1.5% agarose gel. Prior to sequencing, PCR products were cleaned using the PCR purification Kit (Capitalbio, China) to remove unincorporated primers and nucleotides. The purified DNA was sequenced using the ABI Big Dye v3.1 Terminator cycle sequencing kit (Applied Biosystems, USA). The sequencing reaction was carried out with an initial denaturing step of 96°C for 1 min, followed by 25 cycles of 96°C for 10 s, 50°C for 5 s, and 60°C for 4 s. The final reaction products were purified using an ethanol/ammonium acetate precipitation method to remove unincorporated dye terminators, sequencing primers, and residual nucleotides. Denatured samples were analyzed on the ABI 3730XL Genetic Analyzer (Applied Biosystems, USA). Finally, sequence mutations were analyzed using Sequence Variation Analysis v1.2 (Capitalbio, China).

### *CYP2D6* Phenotyping with DM

A total of 235 subjects with *CYP2D6^∗^1/^∗^1* (*n* = 22), *CYP2D6^∗^1/^∗^10* (*n* = 93), *CYP2D6^∗^10/^∗^10* (*n* = 85), and *CYP2D6^∗^5/^∗^10* (*n* = 35) were phenotyped for *CYP2D6* using DM as the probe drug and DX as the *CYP2D6*-specific metabolite. Each subject received 15 mg DM (Tylenol Cold Tablet containing DM, Johnson & Johnson Investment, Ltd, Shanghai, China) with 300 ml of water. Venous blood samples and saliva samples were collected 3 h post-drug administration. Urine samples were collected at 0–3 h intervals post-drug administration. Concentrations of DM and unconjugated DX in all samples were analyzed using a sensitive and validated high performance liquid chromatography tandem mass spectrometry (HPLC–MS/MS) assay as previously described (Hou et al., 1991; Hu et al., 1998). The lower limit of quantification was 0.05 ng/mL for both DM and DX in urine, plasma, and saliva samples. A MR of the concentration of DM over DX (MR_DM/DX_) was used as a measure of *CYP2D6* enzyme activity in the three sample types, respectively.

### Statistical Analysis

Data were expressed as mean values ± SD. Based on urinary, plasma, and salivary phenotyping methods, the MR values between different genotype groups were analyzed respectively with an analysis of variance (ANOVA) test. Allele *CYP2D6^∗^1* was defined as a standard full functional allele and *CYP2D6^∗^5* was defined as a non-functional allele. Both *CYP2D6^∗^1* and *CYP2D6^∗^5* were used as references or control alleles to estimate the impact of the *CYP2D6^∗^10* allele on *CYP2D6* metabolic activity. The AS system was measured by assigning scores of 2, 1.5, 1, and 0.5 to subjects with *CYP2D6^∗^1/^∗^1*, *^∗^1/^∗^10*, *^∗^10/^∗^10*, and *^∗^5/^∗^10*, respectively. A *P* value less than 0.05 was considered to be statistically significant. The ANOVA was performed with SPSS (version 19.0, SPSS^TM^).

## Results

### Demographic Characteristics

Of the investigated subjects, 404 subjects were ethnically Han; the remaining 17 subjects included Hui, Manchu, Khalkhas, Korean, Zhuang, Tujia, and Mongol ethnicities. Their ages, weights, body mass index (BMI) and genders can be found in **Table 1**.

**Table 1:** Demographic characteristics of the study population of healthy Chinese volunteers (mean ± SD).

Characteristic	All subjects (*n* = 421)	*CYP2D6^∗^1/^∗^1* (AS = 2) (*n* = 22)	*CYP2D6^∗^1/^∗^10* (AS = 1.5) (*n* = 93)	*CYP2D6^∗^10/^∗^10* (AS = 1) (*n* = 85)	*CYP2D6^∗^5/^∗^10* (AS = 0.5) (*n* = 35)
Age (years)	30.6 ± 8.5	31.1 ± 7.0	29.0 ± 7.1	31.8 ± 9.6	30.5 ± 6.5
Weight (kg)	65.1 ± 8.5	68.2 ± 10.8	65.0 ± 8.6	63.6 ± 8.1	64.2 ± 7.6
BMI (kg/m^2^)	23.2 ± 2.4	24.1 ± 2.6	22.9 ± 2.4	22.7 ± 2.5	23.1 ± 2.0
Gender	Male = 295	Male = 16	Male = 67	Male = 58	Male = 25
	Female = 126	Female = 6	Female = 26	Female = 27	Female = 10

*BMI, body mass index; AS, activity score*.

### *CYP2D6* Genotypes

Among the 421 subjects, the gene frequency of *CYP2D6^∗^10* was 45.7%. There were 22 subjects with *CYP2D6^∗^1/^∗^1*, 93 subjects with *CYP2D6^∗^1/^∗^10*, 85 subjects with *CYP2D6^∗^10/^∗^10*, and 35 subjects with *CYP2D6^∗^5/^∗^10*. The *CYP2D6^∗^10* allele was also heterozygous with other alleles besides *^∗^1* and *^∗^5* although those genotypes were not reported in this study. Other *CYP2D6* alleles were tested to determine *CYP2D6^∗^1, ^∗^5*, and *^∗^10* specifically and remove the confounding factors.

### *CYP2D6* Phenotypes

Based on the MR values from the 0 to 3 h urine samples, the mean MR in 235 subjects was 0.485 ± 2.48, ranging from 0.00758 to 36.7, and displayed greater than 4,800-fold inter-individual range. Based on the MR values from the 3 h plasma samples, the mean MR in 235 subjects was 1.73 ± 6.63, ranging from 0.0528 to 95.6, and displayed greater than 1800-fold inter-individual range. Based on the MR values from the 3 h saliva samples, the mean MR in 235 subjects was 5.24 ± 18.8, ranging from 0.117 to 250, and displayed greater than 2100-fold inter-individual range.

**FIGURE 1:**
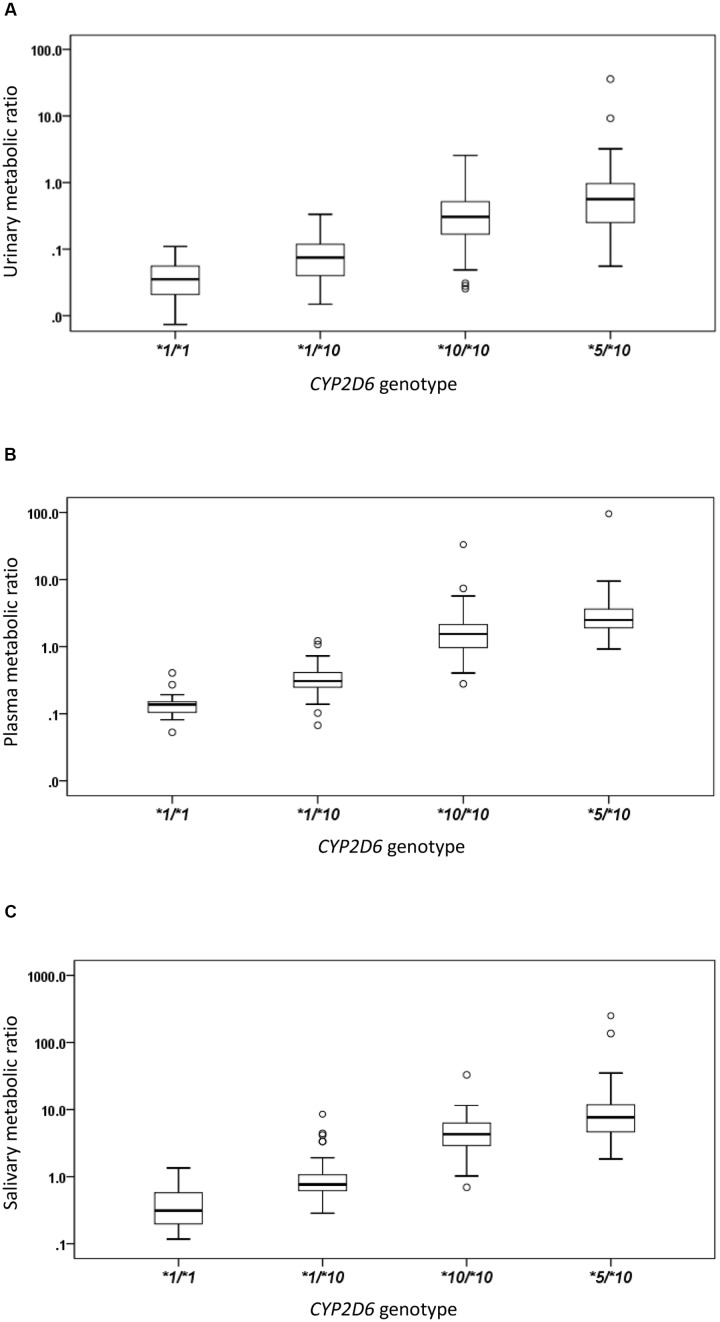
**The box plots of metabolic ratio (MR) in the wild type (*CYP2D6^∗^1/^∗^1*) and the three *CYP2D6^∗^10* allele relevant genotypes in 235 healthy Chinese subjects**. The y-axis is logarithmic and the ratio is based on the MR_DM/DX_. **(A)** Urinary MR; **(B)** plasma MR; **(C)** salivary MR. Box plot explanation: upper horizontal line of box, 75th percentile; lower horizontal line of box, 25th percentile; horizontal bar within box, median; upper horizontal bar outside box, 95th percentile; lower horizontal bar outside box, 5th percentile. Circles represent outliers.

The *CYP2D6^∗^10* allele had substantial impact on the metabolic activity of *CYP2D6* regardless of the urinary, plasma, or salivary phenotyping method used. The mean (± SD) MRs for *CYP2D6^∗^1/^∗^1, ^∗^1/^∗^10, ^∗^10/^∗^10*, and *^∗^5/^∗^10* groups were presented by the three methods, respectively (**Table 2** and **Figure 1**). The urinary, plasma, or salivary MRs increased successively in subjects with *CYP^∗^1/^∗^1, ^∗^1/^∗^10, ^∗^10/^∗^10*, and *^∗^5/^∗^10* with statistical significance (all *P*-values < 0.001) (**Table 2**).

**Table 2:** The MRs for *CYP2D6^∗^1/^∗^1*, *^∗^1/^∗^10*, *^∗^10/^∗^10*, and *^∗^5/^∗^10* groups based on the three phenotyping methods.

	*CYP2D6^∗^1/^∗^1*	*CYP2D6^∗^1/^∗^10*	*CYP2D6^∗^10/^∗^10*	*CYP2D6^∗^5/^∗^10*	*P*-value	*P*-value	*P*-value	*P*-value
	(AS = 2)	(AS = 1.5)	(AS = 1)	(AS = 0.5)	(IM vs. EM)	(*^∗^10/^∗^10* vs. *^∗^1/^∗^10*)	(*^∗^10/^∗^10* vs. *^∗^1/^∗^1*)	(*^∗^1/^∗^10* vs. *^∗^1/^∗^1*)


	(*n* = 22)	(*n* = 93)	(*n* = 85)	(*n* = 35)				
Urinary MR	0.0437 ± 0.0289	0.0915 ± 0.0661	0.420 ± 0.408	1.96 ± 6.25	<0.001	<100.0	<0.001	0.001
Plasma MR	0.146 ± 0.0730	0.351 ± 0.180	2.03 ± 3.57	5.63 ± 15.7	<0.001	<100.0	0.015	<0.001
Salivary MR	0.427 ± 0.308	1.05 ± 1.06	5.09 ± 3.95	19.8 ± 45.9	<0.001	<100.0	<0.001	0.007

*AS, activity score*.

### Correlations among Urinary, Plasma, and Salivary MR

**FIGURE 2:**
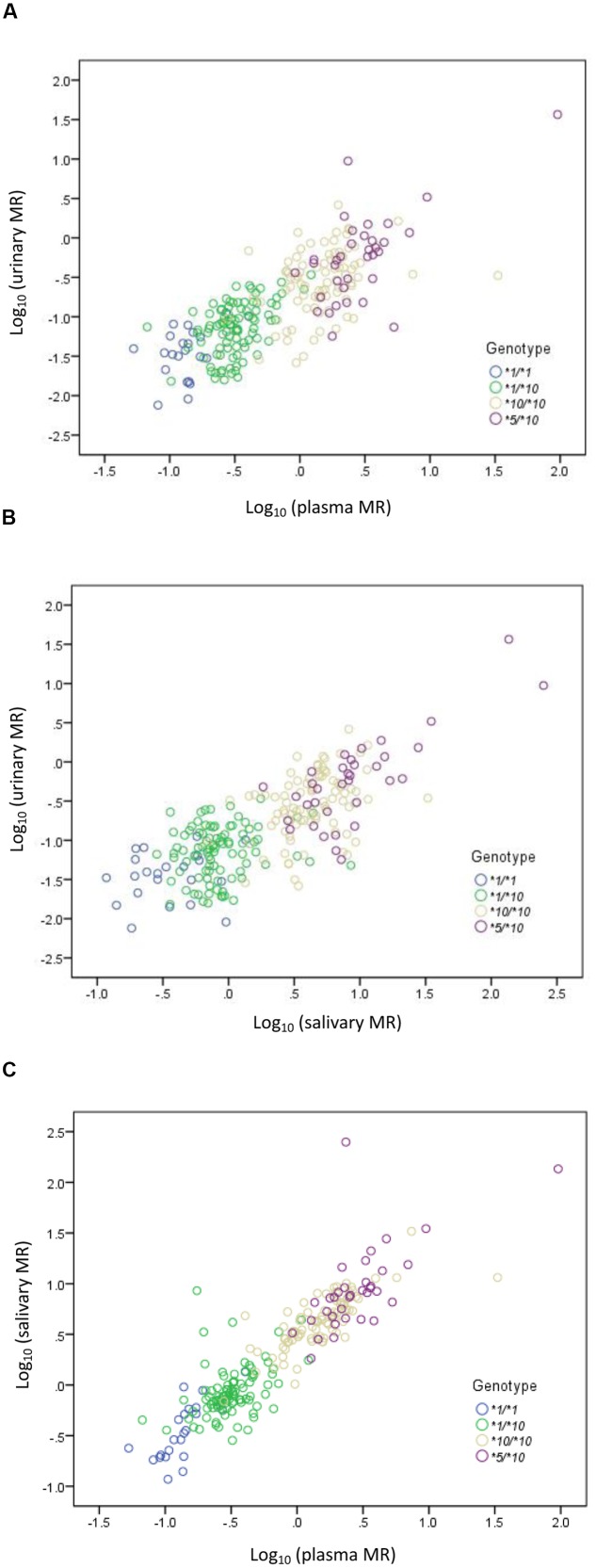
**Correlations among urinary, plasma, and salivary MRs in *CYP2D6^∗^1/^∗^1*, *^∗^1/^∗^10*, *^∗^10/^∗^10*, and *^∗^5/^∗^10* genotypes, respectively, in double logarithmic coordinates. (A)** Correlation between urinary MR and plasma MR (Spearman’s correlation coefficient = 0.780 with *P* < 0.001); **(B)** correlation between urinary MR and salivary MR (Spearman’s correlation coefficient = 0.717 with *P* < 0.001); **(C)** correlation between plasma MR and salivary MR (Spearman’s correlation coefficient = 0.903 with *P* < 0.001).

A statistically significant correlation was found between urinary MR and plasma MR. The Spearman’s correlation coefficient measuring the statistical dependence between the ranking of two variables was 0.780 (*P* < 0.001) (**Figure 2A**). A statistically significant correlation was also found between urinary MR and salivary MR and the Spearman’s correlation coefficient was 0.717 (*P* < 0.001) (**Figure 2B**). Similarly, the correlation between plasma MR and salivary MR was also statistically significant and the Spearman’s correlation coefficient was 0.903 (*P* < 0.001) (**Figure 2C**).

## Discussion

The *CYP2D6^∗^10* variant is the most frequent allele in Asians and a large sample study revealed that *CYP2D6^∗^10* is the most common allele (42.6%) in the Chinese Han population, followed by *CYP2D6^∗^1* (26.5%) (Qian et al., 2013). It is well-known that *CYP2D6^∗^10* is a reduced function allele and it decreases the enzyme activity of *CYP2D6*. The present study was conducted to investigate the impact of the *CYP2D6^∗^10* allele on *CYP2D6* metabolic activity. To exclude confounding factors, we defined *CYP2D6^∗^1* as the only full functional allele and *CYP2D6^∗^5* as the only non-functional allele. Both alleles were used as controls to investigate the impact of *CYP2D6^∗^10* in different allelic combinations.

The urinary, plasma, or salivary MR values increased successively in subjects with *CYP2D6^∗^1/^∗^1, ^∗^1/^∗^10, ^∗^10/^∗^10*, or *^∗^5/^∗^10* (all *P* < 0.001). According to the Clinical Pharmacogenetics Implementation Consortium (CPIC) guideline, subjects with *CYP2D6^∗^1/^∗^1*, *^∗^1/^∗^10*, and *^∗^10/^∗^10* are classified as NMs, and subjects with *CYP2D6^∗^5/^∗^10* are classified as IMs (Hicks et al., 2016). In the present study, MR values in IMs were significantly higher than those in NMs as expected, regardless of the urinary, plasma, or salivary phenotyping method used (all *P* < 0.001). In the NM group, however, a large diversity in MR values was still observed. Although *CYP2D6^∗^1/^∗^10* and *CYP2D6^∗^10/^∗^10* were both classified as NMs similar to the designation given to *CYP2D6^∗^1/^∗^1*, the enzyme activity for subjects carrying *CYP2D6^∗^10* differed to a great extent from the wild type due to the reduced function allele. Both *CYP2D6^∗^1/^∗^10* and *^∗^10/^∗^10* had predicted different enzyme activities as compared with *^∗^1/^∗^1*. Regardless of which phenotyping method was used, the MRs of subjects with *CYP2D6^∗^10/^∗^10* were significantly higher than those of subjects with *CYP2D6^∗^1/^∗^10* and *CYP2D6^∗^1/^∗^1*, respectively, and the MRs of subjects with *CYP2D6^∗^1/^∗^10* were significantly higher than those of subjects with *CYP2D6^∗^1/^∗^1* (all *P* < 0.05). Homozygous *CYP2D6^∗^10/^∗^10* could decrease the *CYP2D6* enzyme activity further than heterozygous *CYP2D6^∗^1/^∗^10*. The mean MR was about 4.6-fold higher in subjects with *CYP2D6^∗^10/^∗^10* than in subjects with *CYP2D6^∗^1/^∗^10* based on the urinary phenotyping method, and the corresponding folds were 5.8 for the plasma method and 4.8 for the salivary method. In comparison, the mean MR was about 4.7-, 2.8-, and 3.9-fold higher in subjects with *CYP2D6^∗^5/^∗^10* than in subjects with *CYP2D6^∗^10/^∗^10* based on urinary, plasma, and salivary phenotyping methods, respectively. The difference between *CYP2D6^∗^1/^∗^10* and *^∗^10/^∗^10* was comparable or even larger than that between *CYP2D6^∗^10/^∗^10* and *^∗^5/^∗^10* and can be seen in **Figure 1**. Large overlaps were observed between *CYP2D6^∗^10/^∗^10* and *^∗^5/^∗^10* as shown in **Figure 2**. Therefore, the NM group calls for a more detailed classification as the large diversity in the NM group could result in an inaccurate prediction of *CYP2D6* enzyme activity. As discussed previously, the *CYP2D6^∗^10/^∗^10* would be more appropriately classified as IM than NM. Our additional analysis showed that two subjects with *CYP2D6^∗^5/^∗^5* had a plasma MR ratio > 70 suggesting that the *^∗^5/^∗^10* individuals are distinct from PMs.

Gaedigk et al. (2008) introduced an AS system for *CYP2D6*. It has since gained acceptance among the scientific community and has been adopted by CPIC (Hicks et al., 2016). This system, in essence, assigns each allele a value that approximates its function, and the sum of the values assigned to each allele is the final AS. Functional alleles with activity levels comparable to the *CYP2D6^∗^1* reference allele are given a value of 1 (Gaedigk et al., 2008). Reduced function and non-functional alleles receive values of 0.5 and 0, respectively (Gaedigk et al., 2008). Gene duplications score double the value given for their single counterparts (Gaedigk et al., 2008). To date, few studies have evaluated the AS system in large Asian populations. The present study with a large sample size indicated that the AS system could benefit from further classification of the NM group, e.g., to distinguish *CYP2D6^∗^1/^∗^10* and *^∗^10/^∗^10*. When classifying the subjects using the AS system, the urinary, plasma, or salivary MR values increased successively from a score of 2 to a score of 0.5 (all *P* < 0.001). The AS system could further classify the NM group into AS 2, 1.5, and 1. On the other hand, the diversity of enzyme activity was observed when evaluating the AS system in Caucasian populations. Hertz et al. (2015) performed a linear regression model and found that the relative activities of a UM, IM, and PM alleles were 0.85, 0.67, and 0.52, respectively, when setting the activity of *CYP2D6^∗^1* at 1.0. The *^∗^2* and *^∗^35* alleles were also assessed as NM alleles. The *^∗^2* allele had an estimated scaled activity of 0.63 while the *^∗^35* allele had an activity of 1.03 (Hertz et al., 2015). The relatively large range of activity observed among *CYP2D6* alleles was not unexpected given the wide range enzyme activities of *CYP2D6*. The current systems for translating *CYP2D6* genotypes into phenotypes are not optimally calibrated (Hertz et al., 2015). The AS system is an adequate classification method although it would be justified to revise the ASs for different alleles for a more accurate prediction of enzyme activities from *CYP2D6* genotypes. Tamoxifen was used as the probe drug in Hertz et al.’s (2015) study and there might be substrate-specific differences among alleles toward different substrates. Other covariates like age and ethnicity should also be taken into account when exploring the extent of variation among phenotypes using genotype information.

Urine sample collection for 8 h is specific and a demanding process for test patients. A more lenient urine sample collection for 4 h or overnight post-drug administration has also been used in clinical studies to alleviate such a strict demand time (Chladek et al., 2000). DX could further conjugate with glucuronide (Duche et al., 1993), so the generated DX was the sum of conjugated and unconjugated DX. Several studies have compared the urinary MRs of β-glucuronidase-treated samples with those of untreated samples and found that the DM/free-DX ratio from untreated samples correlated well with the DM/total-DX ratio, although the absolute values of the two ratios may differ (Duche et al., 1993). Therefore, the DM/free-DX ratio can also be used to determine the phenotype. Urinary MR based on free compounds has been developed for phenotyping (Yeh et al., 2003). Plasma and salivary phenotyping methods have also been developed for simple and robust *CYP2D6* phenotyping (Frank et al., 2007). Our previous studies indicate that MR from single-point plasma from 1 to 30 h after a single dose of DM could predict the MR from AUC well and could be used as the *CYP2D6* phenotyping method for NMs, IMs, and PMs (Chen et al., 2016a,b). In consideration of clinical convenience and the urinary sample collection intervals, plasma and saliva samples were taken 3 h post-administration while urinary samples were collected at 0–3 h intervals. Few studies have explored the correlations among urinary, plasma, and salivary phenotyping methods simultaneously in large sample sizes. In the present study, statistically significant pair-wise correlations were found between any two of the three phenotyping methods (*P* < 0.001). Urinary MR appeared to have a smaller correlation coefficient than plasma and salivary MRs. This trend was also seen in previous studies (Hu et al., 1998; Chen et al., 2016b) and may be due to the fact that plasma and salivary MRs were collected from a single time point while urine samples were collected over a range of time. The large sample size confirmed that urinary, plasma, and salivary MRs were highly correlated and alternative phenotyping methods could be used for clinical convenience. This result was consistent with data previously reported from small samples (Chladek et al., 2000; Frank et al., 2007; Chen et al., 2016a,b). The 8-h urinary phenotyping method is used widely around the world. In clinical practice, this procedure may work well for inpatients but is inconvenient for outpatients who have to deliver the urinary samples at a later time. Plasma and salivary methods are good for saving time although plasma samples may be inconvenient to collect. The salivary method is especially convenient for sample collections in children.

The DM/free-DX ratio and DM/total-DX ratio could both be used for *CYP2D6* phenotyping although the absolute values of the two ratios may differ. Only unconjugated DX was found in saliva, and therefore the DM/free-DX ratio was used for all three sample types in this study. The widely accepted anti-mode of DM/total-DX of 0.3 used to identify PMs was not applicable here. To exclude confounding factors, a strict definition of the standard full functional allele (*CYP2D6^∗^1*) caused the wild type number with two full functional alleles to be relatively small in the present study. The frequency of PM is very low (0–2%) in the Chinese population (Gaedigk, 2013) and therefore the MR values of *CYP2D6^∗^5/^∗^5* were not evaluated here. Large overlaps of MR values between adjacent genotypes were clearly observed and created an insurmountable problem when predicting phenotypes from *CYP2D6* genotypes. There were not convincible explanations for some outliers observed in the present study resulting in difficulty applying genotype information to individual patients. It was difficult to know if an individual carrying the variant of interest will display the predicted phenotype (Leeder and Gaedigk, 2014).

## Conclusion

The urinary, plasma, or salivary MR values increased successively in subjects with *CYP2D6^∗^1/^∗^1*, *^∗^1/^∗^10*, *^∗^10/^∗^10*, or *^∗^5/^∗^10* genotypes (all *P* < 0.001). In the NM group, homozygous *CYP2D6^∗^10/^∗^10* showed a greater decrease in *CYP2D6* enzyme activity than the heterozygous *CYP2D6^∗^1/^∗^10* genotype. The NM group calls for a more detailed classification due to its broad range of MR values. The AS system has shown to be a more accurate measurement for enzyme activity prediction compared to grouping a number of genotypes into a single phenotype group. Urinary, plasma, and salivary MRs were highly correlated and the latter two could be used as alternative phenotyping methods for clinical convenience.

## Author Contributions

RC designed and preformed the study. XZ analyzed the samples. PH designed the study.

## Conflict of Interest Statement

The authors declare that the research was conducted in the absence of any commercial or financial relationships that could be construed as a potential conflict of interest.

## Footnotes

1 http://www.cypalleles.ki.se/

## References

[B1] Caudle K. E. Dunnenberger H. M. (2017). Standardizing terms for clinical pharmacogenetic test results: consensus terms from the Clinical Pharmacogenetics Implementation Consortium (CPIC). *Genet. Med.* 19 215–223. 10.1038/gim.2016.87 27441996 PMC5253119

[B2] Chen R. Rostami-Hodjegan A. Wang H. Berk D. Shi J. Hu P. (2016a). Application of a physiologically based pharmacokinetic model for the evaluation of single-point plasma phenotyping method of CYP2D6. *Eur. J. Pharm. Sci.* 92 131–136. 10.1016/j.ejps.2016.07.001 27412587

[B3] Chen R. Wang H. Shi J. Hu P. (2016b). Alternative methods for CYP2D6 phenotyping: comparison of dextromethorphan metabolic ratios from AUC, single point plasma, and urine. *Int. J. Clin. Pharmacol. Ther.* 54 330–336. 10.5414/CP202387 26902503

[B4] Chladek J. Zimova G. Beranek M. Martinkova J. (2000). In-vivo indices of CYP2D6 activity: comparison of dextromethorphan metabolic ratios in 4-h urine and 3-h plasma. *Eur. J. Clin. Pharmacol.* 56 651–657. 10.1007/s002280000218 11214771

[B5] Duché J. C. Quérol-Ferrer V. Barré J. Mésangeau M. Tillement J. P. (1993). Dextromethorphan O-demethylation and dextrorphan glucuronidation in a French population. *Int. J. Clin. Pharmacol. Ther. Toxicol.* 31 392–398. 8225685

[B6] Frank D. Jaehde U. Fuhr U. (2007). Evaluation of probe drugs and pharmacokinetic metrics for CYP2D6 phenotyping. *Eur. J. Clin. Pharmacol.* 63 321–333. 10.1007/s00228-006-0250-8 17273835

[B7] Gaedigk A. (2013). Complexities of CYP2D6 gene analysis and interpretation. *Int. Rev. Psychiatry* 25 534–553. 10.3109/09540261.2013.825581 24151800

[B8] Gaedigk A. Sangkuhl K. Whirl-Carrillo M. Klein T. Leeder J. S. (2017). Prediction of CYP2D6 phenotype from genotype across world populations. *Genet. Med.* 19 69–76. 10.1038/gim.2016.80 27388693 PMC5292679

[B9] Gaedigk A. Simon S. Pearce R. Bradford L. Kennedy M. Leeder J. (2008). The CYP2D6 activity score: translating genotype information into a qualitative measure of phenotype. *Clin. Pharmacol. Ther.* 83 234–242. 10.1038/sj.clpt.6100406 17971818

[B10] Hersberger M. Marti-Jaun J. Rentsch K. Hanseler E. (2000). Rapid detection of the CYP2D6^∗^3, CYP2D6^∗^4, and CYP2D6^∗^6 alleles by tetra-primer PCR and of the CYP2D6^∗^5 allele by multiplex long PCR. *Clin. Chem.* 46 1072–1077. 10926885

[B11] Hertz D. L. Snavely A. C. Mcleod H. L. Walko C. M. Ibrahim J. G. Anderson S. (2015). In vivo assessment of the metabolic activity of CYP2D6 diplotypes and alleles. *Br. J. Clin. Pharmacol.* 80 1122–1130. 10.1111/bcp.12665 25907378 PMC4631184

[B12] Hicks J. K. Sangkuhl K. Swen J. J. Ellingrod V. L. Müller D. J. Shimoda K. (2016). Clinical Pharmacogenetics Implementation Consortium guideline (CPIC) for CYP2D6 and CYP2C19 genotypes and dosing of tricyclic antidepressants: 2016 Update. *Clin. Pharmacol. Ther.* 10.1002/cpt.597 [Epub ahead of print]. PMC5478479 27997040

[B13] Hou Z. Y. Pickle L. W. Meyer P. S. Woosley R. L. (1991). Salivary analysis for determination of dextromethorphan metabolic phenotype. *Clin. Pharmacol. Ther.* 49 410–419. 10.1038/clpt.1991.48 2015730

[B14] Hu O. Y. Tang H. S. Lane H. Y. Chang W. H. Hu T. M. (1998). Novel single-point plasma or saliva dextromethorphan method for determining CYP2D6 activity. *J. Pharmacol. Exp. Ther.* 285 955–960. 9618394

[B15] Ito T. Kato M. Chiba K. Okazaki O. Sugiyama Y. (2010). Estimation of the interindividual variability of cytochrome 2D6 activity from urinary metabolic ratios in the literature. *Drug Metab. Pharmacokinet.* 25 243–253. 10.2133/dmpk.25.243 20610883

[B16] Kitada M. (2002). Genetic polymorphism of cytochrome P450 enzymes in Asian populations: focus on CYP2D6. *Int. J. Clin. Pharmacol. Res.* 23 31–35. 14621071

[B17] Leeder J. S. Gaedigk A. (2014). CYP2D6 and pharmacogenomics: where does future research need to focus? Part 2: clinical aspects. *Pharmacogenomics* 15 1055–1058. 10.2217/pgs.14.27 25084197

[B18] Lötsch J. Rohrbacher M. Schmidt H. Doehring A. Brockmöller J. Geisslinger G. (2009). Can extremely low or high morphine formation from codeine be predicted prior to therapy initiation? *Pain* 144 119–124. 10.1016/j.pain.2009.03.023 19395173

[B19] Løvlie R. Daly A. K. Molven A. Idle J. R. Steen V. M. (1996). Ultrarapid metabolizers of debrisoquine: characterization and PCR-based detection of alleles with duplication of the CYP2D6 gene. *FEBS Lett.* 392 30–34. 10.1016/0014-5793(96)00779-X 8769309

[B20] Murphy E. McMahon F. J. (2013). Pharmacogenetics of antidepressants, mood stabilizers, and antipsychotics in diverse human populations. *Discov. Med.* 16 113–122. 23998447 PMC6011657

[B21] O’mathúna B. Farré M. Rostami-Hodjegan A. Yang J. Cuyàs E. Torrens M. (2008). The consequences of 3, 4-methylenedioxymethamphetamine induced CYP2D6 inhibition in humans. *J. Clin. Psychopharmacol.* 28 523–529. 10.1097/JCP.0b013e318184ff6e 18794647

[B22] Qian J. C. Xu X. M. Hu G. X. Dai D. P. Xu R. A. Hu L. M. (2013). Genetic variations of human CYP2D6 in the Chinese Han population. *Pharmacogenomics* 14 1731–1743. 10.2217/pgs.13.160 24192122

[B23] Teh L. K. Bertilsson L. (2012). Pharmacogenomics of CYP2D6: molecular genetics, interethnic differences and clinical importance. *Drug Metab. Pharmacokinet.* 27 55–67. 10.2133/dmpk.DMPK-11-RV-121 22185816

[B24] Yeh G. C. Tao P. L. Ho H. O. Lee Y. J. Chen J. Y. Sheu M. T. (2003). Analysis of pharmacokinetic parameters for assessment of dextromethorphan metabolic phenotypes. *J. Biomed. Sci.* 10 552–564. 10.1159/000072383 12928596

[B25] Zanger U. M. Schwab M. (2013). Cytochrome P450 enzymes in drug metabolism: regulation of gene expression, enzyme activities, and impact of genetic variation. *Pharmacol. Ther.* 138 103–141. 10.1016/j.pharmthera.2012.12.007 23333322

[B26] Zhou S. F. (2009a). Polymorphism of human cytochrome P450 2D6 and its clinical significance: part I. *Clin. Pharmacokinet.* 48 689–723. 10.2165/11318030-000000000-00000 19817501

[B27] Zhou S. F. (2009b). Polymorphism of human cytochrome P450 2D6 and its clinical significance: part II. *Clin. Pharmacokinet.* 48 761–804. 10.2165/11318070-000000000-00000 19902987

