# Supplementary material for: CYP2D6 Phenotyping Using Urine, Plasma, and Saliva Metabolic Ratios to Assess the Impact of CYP2D6∗10 on Interindividual Variation in a Chinese Population
Source: Front Pharmacol. 2017 May 2;8:239. doi: 10.3389/fphar.2017.00239 (PMC5411458; doi:10.3389/fphar.2017.00239)
Supplement: Supplementary file 1 [file Table_1.docx]

| Primer | Sequence |
| --- | --- |
| CYP2D6-N1-F1 | CGCTCGGTGTGCTGAGAGTGT |
| CYP2D6-N2-F1 | CTGGGAATGGGATGCTAACT |
| CYP2D6-N3-F1 | GGAGTGGGTGGTGGATGGT |
| CYP2D6-N4-F1 | TACCTCCTATCCACGTCAGAG |
| CYP2D6-N5-F1 | TTCTGTCCCGAGTATGCTCTC |
| CYP2D6-N6-F1 | TGTCCCCCGTGTGTTTGGT |
| CYP2D6-W1-F | GTTATCCCAGAAGGCTTTGCAGGCTTCA |
| CYP2D6-W1-R | CCCTCCTGTGCTCTGCGTTCACCTGGACAAG |
| CYP2D6-W2-F | CAAGGACTCTGTACCTCCTATCCACGTCAG |
| CYP2D6-W2-R | GAGGGTGGTGGCTCAGTCCTGGGCTTCCAT |

Table 1: The primers for CYP2D6 sequencing
